# Clinical Applications of the ARDSVet (Acute Respiratory Distress Syndromes in Veterinary Medicine) Definitions in Small and Large Animal Patients

**DOI:** 10.1111/vec.70015

**Published:** 2025-09-11

**Authors:** Tereza Stastny, Daniela Bedenice, JoAnn Slack, Anusha Balakrishnan, Deborah C. Silverstein

**Affiliations:** ^1^ Department of Clinical Sciences and Advanced Medicine, School of Veterinary Medicine University of Pennsylvania Philadelphia Pennsylvania USA; ^2^ Department of Clinical Sciences Cummings School of Veterinary Medicine at Tufts University North Grafton Massachusetts USA; ^3^ Department of Clinical Studies, New Bolton Center, School of Veterinary Medicine University of Pennsylvania Kennett Square Pennsylvania USA; ^4^ Veterinary Emergency Group White Plains New York USA

**Keywords:** clinical guidelines, consensus, hypoxemia, lung injury, respiratory failure

## Abstract

**Objective:**

To illustrate the use of the ARDSVet (Acute Respiratory Distress Syndromes in Veterinary Medicine) definitions in small and large animal patients using a case‐based approach.

**Etiology:**

Acute respiratory distress syndrome (ARDS) in veterinary patients is triggered by a wide range of clinical insults. These include probable risk factors such as systemic inflammation, pancreatitis, and sepsis, as well as possible risk factors such as blood transfusions and ventilator‐induced lung injury. These conditions may lead to diffuse alveolar damage and increased pulmonary capillary permeability.

**Diagnosis:**

ARDS remains challenging to diagnose, particularly in veterinary patients with variable resources. The updated ARDSVet definitions offer a structured framework based on five criteria: a known or suspected risk factor, onset of respiratory distress within 1 week, exclusion of cardiogenic edema and volume overload, thoracic imaging (including point‐of‐care ultrasound) demonstrating diffuse pulmonary infiltrates, and impaired oxygenation assessed by PaO_2_/FiO_2_ or SpO_2_/FiO_2_ ratios. Case vignettes highlight revised oxygenation thresholds, expanded use of point‐of‐care ultrasound, and the role of advanced respiratory support techniques.

**Therapy:**

ARDS treatment is primarily supportive, focusing on oxygen supplementation, high‐flow nasal oxygen, and/or mechanical ventilation, along with management of the underlying cause. While ARDSVet does not offer formal treatment guidelines, case vignettes illustrate how supportive strategies may be adapted across disease stages without endorsing any specific therapeutic interventions.

**Prognosis:**

Prognosis in animals with ARDS is influenced by the severity of respiratory compromise, the underlying cause, and the timelines of appropriate interventions. The updated definitions will aid clinicians in early and timely recognition of ARDS, although further studies are needed to assess its impact on clinical outcomes.

**Abbreviations**
ARDSVet: Acute Respiratory Distress Syndromes in Veterinary Medicine
HFNO: high‐flow nasal oxygen
IMV: invasive mechanical ventilation
LA:Ao: left atrium‐to‐aorta
L‐CHF: left‐sided congestive heart failure
PEEP: positive end‐expiratory pressure
POCUS: point‐of‐care ultrasound
SpO_2_
: hemoglobin oxygen saturation determined by pulse oximetry

## 1 Introduction

Following the development of the updated Acute Respiratory Distress Syndromes in Veterinary Medicine (ARDSVet) definitions for ARDS in veterinary medicine [1], the veterinary ARDS Working Group created a series of clinical vignettes to illustrate how these definitions can be applied in both small and large animal patients at risk for, or with, ARDS. The ARDSVet consensus definitions include detailed domain descriptions supported by evidence summaries that informed the final consensus [1]. This case‐based manuscript aims to provide additional guidance on the clinical implementation of the ARDSVet definitions.

Case descriptions are intentionally succinct, focusing on key updates in the ARDSVet definitions, particularly where they differ from the original veterinary ARDS definitions [2]. These updates include revised oxygenation criteria, the use of point‐of‐care ultrasound (POCUS), and the implementation of advances in supportive care, such as high‐flow nasal oxygen (HFNO) and mechanical ventilation, as outlined in Domains 2 and 3. Where applicable, risk factors and population‐specific considerations from Domain 1 are also included.

Given the variability in veterinary practice settings, emphasis was placed on available diagnostic and therapeutic modalities, including considerations for resource‐limited environments, paralleling the Kigali Modification of the Berlin Definition in human ARDS [3]. These case vignettes were designed to also illustrate the application of the ARDSVet definitions across the continuum of disease progression. The therapeutic approaches discussed in these examples are intended to showcase disease progression and application of the definitions across different stages of the disease, rather than endorsing any specific therapies for veterinary ARDS.

Clinicians are encouraged to adapt these guidelines based on locally available resources and patient‐specific needs.

## 2 Case Scenario 1: A Dog With Severe Pancreatitis That Develops Respiratory Distress

### 2.1 Domain 1: Defining Populations at Risk and Identifying Risk Factors for ARDS

#### 2.1.1 History and Physical Exam

A 5‐year‐old male neutered Miniature Schnauzer (6 kg) presents with vomiting and acute abdominal pain and is diagnosed with severe acute pancreatitis on abdominal ultrasound. After 48 h of hospitalization, the dog develops progressive tachypnea with a respiratory rate of 60/min, increased respiratory effort, and a restrictive breathing pattern. Thoracic auscultation reveals bilaterally increased lung sounds. The patient is placed in an oxygen kennel (FiO_2_ 60%) for respiratory support.

#### 2.1.2 Application of Domain 1

Pancreatitis is a well‐documented “probable” risk factor for ARDS in dogs, specifically when diagnosed within 1 week of new or worsening respiratory distress (Working Group Recommendations 1.3, Table 1). This patient falls into an at‐risk population due to the temporal association between severe acute pancreatitis and the onset of tachypnea 48 h later. Close monitoring and further diagnostics (see Domain 2) are warranted to assess for ARDS.

**TABLE 1:** Probable risk factors for veterinary acute respiratory distress syndromes.

Risk factor	Level of agreement among Working Group members
Aspiration injury	9/9
Systemic inflammation	8/9 (1/9 neutral)
Sepsis	9/9
Pancreatitis	9/9
Aerosolized toxin inhalation	9/9
Drowning	7/7
Trauma: Thoracic Extrathoracic	8/9 (1/9 neutral)
Smoke inhalation	9/9
*Rhodococcus equi* infection in horses	8/9 (1/9 neutral)
Equine influenza infection in horses	8/9 (1/9 neutral)

### 2.2 Domain 2: Diagnostic Criteria Used in Small Animals

#### 2.2.1 Diagnostics

**FIGURE 1:**
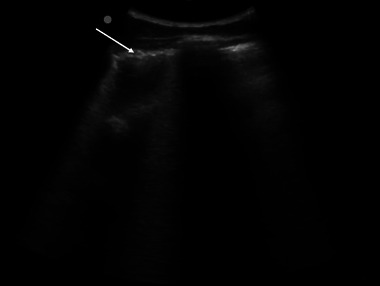
Thoracic point‐of‐care ultrasound of the left dorsal hemothorax depicting coalescing B‐lines and an irregular pleural line. The arrow indicates the irregular pleural line.

**FIGURE 2:**
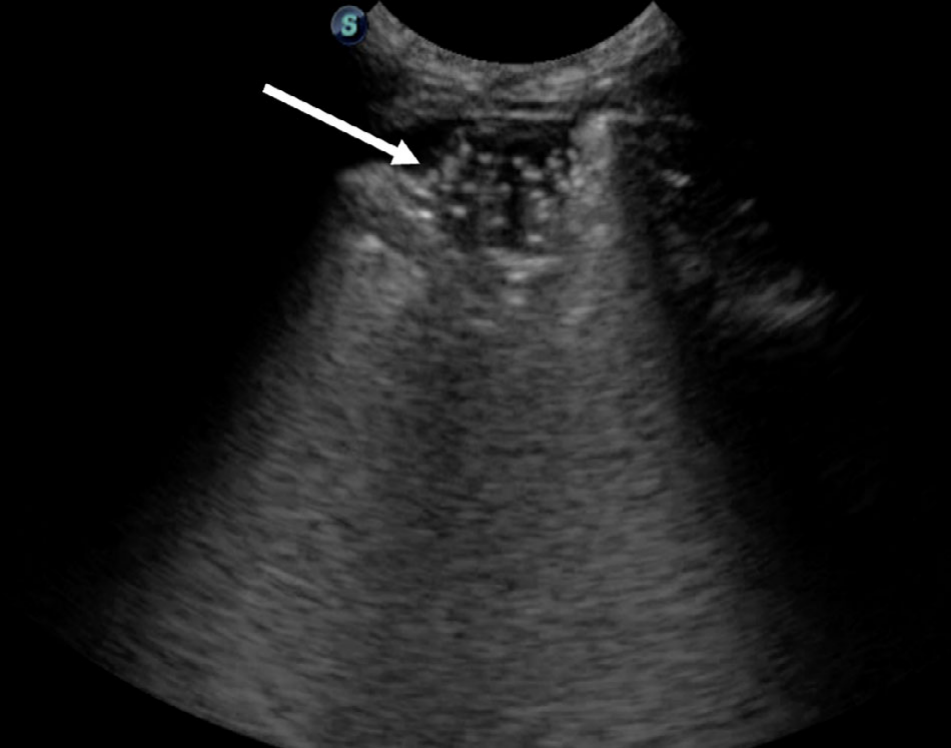
Thoracic point‐of‐care ultrasound of the right mid‐hemothorax depicting a “shred” sign, indicating partial lung consolidation, with a hyperechoic, serrated transition from consolidated to aerated lung. The arrow points to the “shred” sign.

**FIGURE 3:**
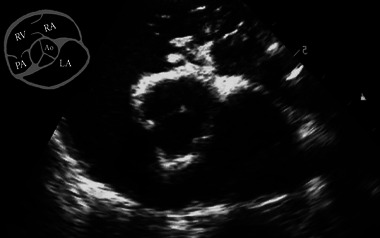
Cardiac point‐of‐care ultrasound of the right parasternal short‐axis view of the heart depicting a normal left atrium‐to‐aorta (LA:Ao) ratio at 1.3:1. The aorta (Ao), left atrium (LA), right atrium (RA), right ventricle (RV), and pulmonary artery (PA) are illustrated in the upper left diagram of the image.

Due to difficulty obtaining an arterial blood sample, oxygenation is assessed using the hemoglobin oxygen saturation determined by pulse oximetry (SpO_2_)/FiO_2_ ratio: SpO_2_ 93%, FiO_2_ 60%, SpO_2_/FiO_2_ ratio 155. Given progressive respiratory distress and increased work of breathing, POCUS is prioritized over thoracic radiographs or computed tomography until the patient stabilizes. Thoracic and cardiac POCUS reveal bilateral coalescing B‐lines, pleural line irregularities, and areas of lung consolidation (shred sign) in the right mid‐hemithorax, consistent with pulmonary infiltrates (Figures 1 and 2). The left atrium‐to‐aorta (LA:Ao) ratio is normal at 1.3:1 (Figure 3), with no pleural effusion or pneumothorax. After 3 h of oxygen supplementation, the patient stabilizes, and thoracic radiographs reveal bilateral, diffuse alveolar infiltrates with a normal heart size.

#### 2.2.2 Application of Domain 2

The patient meets the five criteria outlined in the updated ARDSVet definition (Working Group Recommendations, Table 3):

1. **Risk factor**: Pancreatitis
2. **Timing**: Respiratory distress within 1 week of clinical signs of pancreatitis
3. **Origin of edema**: Left‐sided congestive heart failure (L‐CHF) and fluid overload were ruled out based on normal LA:Ao ratio on POCUS, absence of cardiac history, heart murmur, or clinical signs, and conservative fluid therapy
4. **Thoracic imaging**: POCUS reveals diffuse, bilateral coalescing B‐lines and irregular pleural lines, supporting ARDS due to increased permeability edema
5. **Oxygenation**: SpO_2_/FiO_2_ ratio of 155 (SpO_2_ 93%, FiO_2_ 60%); mild/moderate hypoxemia ARDSVet category for nonintubated ARDS.

This patient meets the oxygenation criterion based on an SpO_2_/FiO_2_ ratio of 155, consistent with mild/moderate hypoxemia (SpO_2_/FiO_2_ ratio >150 and ≤315 with SpO_2_ ≤97% [Working Group Recommendations, Table 3]). While the previous veterinary ARDS definitions used the PaO_2_/FiO_2_ ratio to assess the severity of hypoxemia, the updated definition now includes both the SpO_2_/FiO_2_ and PaO_2_/FiO_2_ ratios to reflect the widespread availability of pulse oximetry in veterinary practice (Working Group Recommendation 2.3b).

Based on POCUS findings, this patient meets the thoracic imaging and origin of edema criteria for ARDSVet. The scan reveals diffuse B‐lines, irregular pleural lines, and partial consolidations, consistent with diffuse pulmonary infiltrates (Working Group Recommendations, Table 3). A normal LA:Ao ratio, absence of cardiac history or abnormalities on physical examination, and conservative fluid administration over the prior 72 h make L‐CHF and volume overload unlikely (Working Group Recommendations, Table 3). These findings are sufficient to fulfill the ARDSVet criteria, although thoracic radiographs later confirm and support this assessment (Working Group Recommendations 2.3c and 2.3d).

### 2.3 Domain 3: Advanced Respiratory Support Therapies in ARDS

#### 2.3.1 Case Progression

The patient's respiratory status worsens. HFNO is initiated at 2 L/kg/min with FiO_2_ 100%. Oxygenation is reassessed: SpO_2_ 88%, FiO_2_ 100%, SpO_2_/FiO_2_ ratio 88.

Six hours later, mechanical ventilation is initiated due to severe hypoxemia and impending respiratory fatigue. The patient is intubated and ventilated with positive end‐expiratory pressure (PEEP) of 8 cm H_2_O. Oxygenation is initially assessed using the SpO_2_/FiO_2_ ratio: SpO_2_ 90%, FiO_2_ 100%, SpO_2_/FiO_2_ ratio 90. Following arterial line placement, the PaO_2_/FiO_2_ ratio is also obtained: PaO_2_ 65 mm Hg, FiO_2_ 100%, PaO_2_/FiO_2_ ratio 65. Forty‐eight hours later, the patient's PEEP is reduced to 6 cm H_2_O and FiO_2_ to 50%. SpO_2_ is 92%, and PaO_2_ is 75 mm Hg, with SpO_2_/FiO_2_ and PaO_2_/FiO_2_ ratios of 184 and 150, respectively.

#### 2.3.2 Application of Domain 3

The updated ARDSVet definitions do not require advanced support, such as invasive mechanical ventilation (IMV) or HFNO, to include animals that otherwise meet ARDS criteria but lack access to such care (Working Group Recommendation 3.3). This patient initially met the criteria for mild/moderate nonintubated ARDS based on supplemental oxygen with a known FiO_2_ (oxygen kennel set at 60%), an SpO_2_/FiO_2_ ratio >150 and ≤315, and fulfillment of the remaining ARDS diagnostic criteria (Working Group Recommendations 3.3c).

As the disease progressed, the patient met criteria for severe nonintubated ARDS, based on HFNO at >1 L/kg/min, an SpO_2_/FiO_2_ ratio ≤150, and continued fulfillment of the remaining diagnostic criteria for nonintubated ARDS (Working Group Recommendations 3.3c).

When mechanical ventilation was initiated, the patient met criteria for severe IMV‐ARDS, based on a PEEP >5 cm H_2_O and an SpO_2_/FiO_2_ ratio ≤150. A subsequent arterial blood gas was also consistent with severe IMV‐ARDS, showing a PaO_2_/FiO_2_ ratio ≤100 (PEEP >5 cm H_2_O). Forty‐eight hours later, the patient transitioned to mild/moderate IMV‐ARDS, with a PEEP >5 cm H_2_O, an SpO_2_/FiO_2_ ratio >150 and ≤315, a PaO_2_/FiO_2_ ratio >100 and ≤300, and continued fulfillment of the diagnostic criteria for IMV‐ARDS (Working Group Recommendations 3.3b).

#### 2.3.3 Summary

This case illustrates key updates in the new ARDSVet definitions compared to the original veterinary ARDS framework. The revised criteria emphasize greater flexibility and accessibility across diverse clinical settings by incorporating SpO_2_/FiO_2_ ratios in addition to PaO_2_/FiO_2_ ratios to assess oxygenation. POCUS is now accepted as a primary imaging modality for assessing pulmonary infiltrates and excluding cardiac causes of edema, particularly when radiographs or computed tomography are not feasible. ARDS severity categories now reflect the use of advanced respiratory support, such as HFNO and mechanical ventilation with a minimum PEEP, without requiring access to these interventions for diagnosis. This case demonstrates how ARDS severity evolves across nonintubated and intubated states while still fulfilling the diagnostic criteria at each stage.

## 3 Case Scenario 2: A Miniature Colt With Aspiration Pneumonia That Progresses to ARDS

### 3.1 Domain 1: Defining Populations at Risk and Identifying Risk Factors for ARDS

#### 3.1.1 History and Physical Exam

A 2‐day old American Miniature Horse colt is presented for progressive lethargy and increasing respiratory effort, starting within 24 h of birth. Parturition at a gestational age of 341 days was uncomplicated without evidence of placental abnormalities. However, the colt failed to meet normal postpartum milestones and required assistance to nurse at home.

**FIGURE 4:**
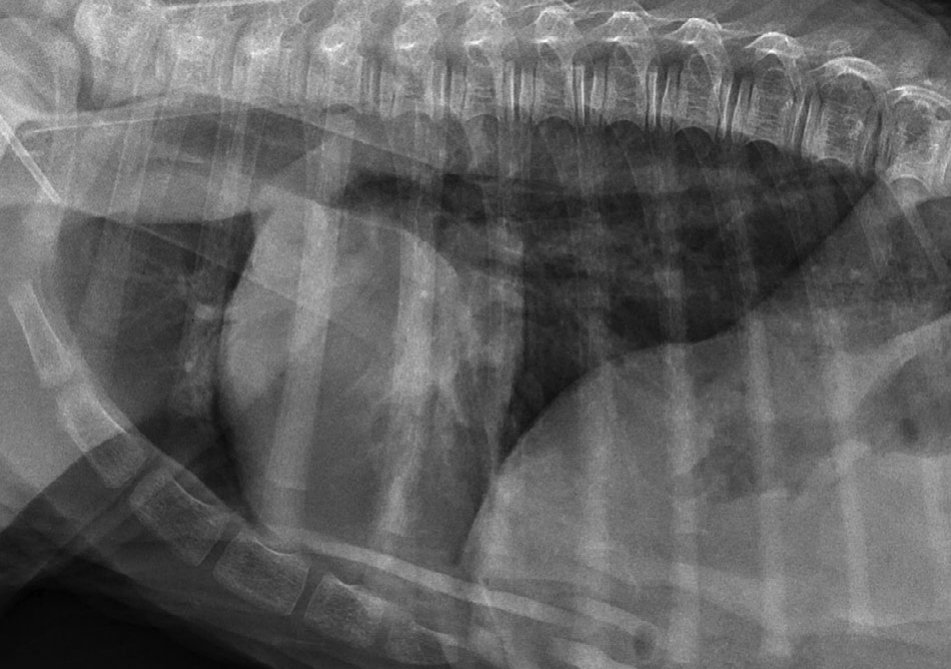
Right lateral thoracic radiograph of a 2‐day‐old American Miniature Horse colt on admission for progressive respiratory distress. A mild alveolar pattern is present in the caudoventral lung, superimposed over the cardiac silhouette. A milder bronchointerstitial lung pattern is noted caudodorsally.

On presentation, the colt was dull, weak, and unable to walk unassisted. Its heart rate and respiratory rate were markedly increased (160/min, reference interval 60–115/min; and 90/min, reference interval 20–40/min, respectively), with a moderate increase in respiratory effort and body temperature (39.6°C [103.2°F], reference interval 37.2°C–38.9°C [99°F–102°F]). Thoracic auscultation while standing showed pronounced lung sounds bilaterally. At this time, the colt was diagnosed with hypovolemia, failure of transfer of passive immunity, suspected sepsis based on clinical evaluation and hematology, and aspiration pneumonia based on thoracic radiographs at admission (Figure 4). Initial arterial blood gas analysis on room air (FiO_2_ 21%) obtained from the dorsal metatarsal artery in temporary lateral recumbency showed moderate hypoxemia (PaO_2_ = 60 mm Hg, reference for 48‐h‐old foals: 74.9 ± 3.3 mm Hg) and hyperventilation (PaCO_2_ = 28 mm Hg, reference: 46.1 ± 1.1 mm Hg) [4].

The patient was hospitalized for intensive care management, including volume support, a plasma transfusion, systemic antimicrobials, and oxygen supplementation.

#### 3.1.2 Application of Domain 1

Aspiration injury and suspected sepsis are well‐documented “probable” risk factors for ARDS, while “transfusion of blood products” is considered a “possible” risk factor (Working Group Recommendations 1.3, Tables 1 and 2). This patient, therefore, falls into an at‐risk population due to the temporal association between acute perinatal illness with aspiration pneumonia and the onset of tachypnea and increased respiratory effort 24–28 h later. Close monitoring and further diagnostics (see Domain 2) are warranted to determine progression of clinical signs and hypoxemia and to recognize the development of ARDS.

**TABLE 2:** Possible risk factors for veterinary ARDS.

Risk factor	Level of agreement among Working Group members
Transfusion of blood products	7/7
Ventilator‐induced lung injury	7/7
*Pneumocystis carinii* infection in horses	6/7 (1/7 neutral)
Other mixed bacterial infections in horses	7/9 (2/9 neutral)
Other viral infections in horses	8/9 (1/9 disagree)

### 3.2 Domain 2: Diagnostic Criteria Used in Large Animals

The ARDSVet definitions propose stratifying veterinary patients into three groups: IMV‐ARDS, nonintubated ARDS, and patients at risk for ARDS (Working Group Recommendations 3.3). The clinical examination, initial thoracic radiographs (Figure 4), and arterial blood gas analysis of this patient did *not* fulfill the diagnostic criteria for ARDSVet at the time of presentation but classified the colt as “at risk for ARDS.” The colt showed moderate hypoxemia with a PaO_2_ of 60 mm Hg but did not meet all other required diagnostic criteria for ARDS due to a lack of generalized pulmonary infiltrates on initial thoracic radiographs.

#### 3.2.1 Case Progression

The patient's respiratory status worsened within 48 h, despite conventional nasal oxygen supplementation (0.2 mL/kg/min). HFNO was thus initiated at 2 L/kg/min with an FiO_2_ of 80%. Oxygenation was reassessed: PaO_2_ = 96 mm Hg, PaCO_2_ = 29 mmHg, PaO_2_/FiO_2_ ratio = 120.

**FIGURE 5:**
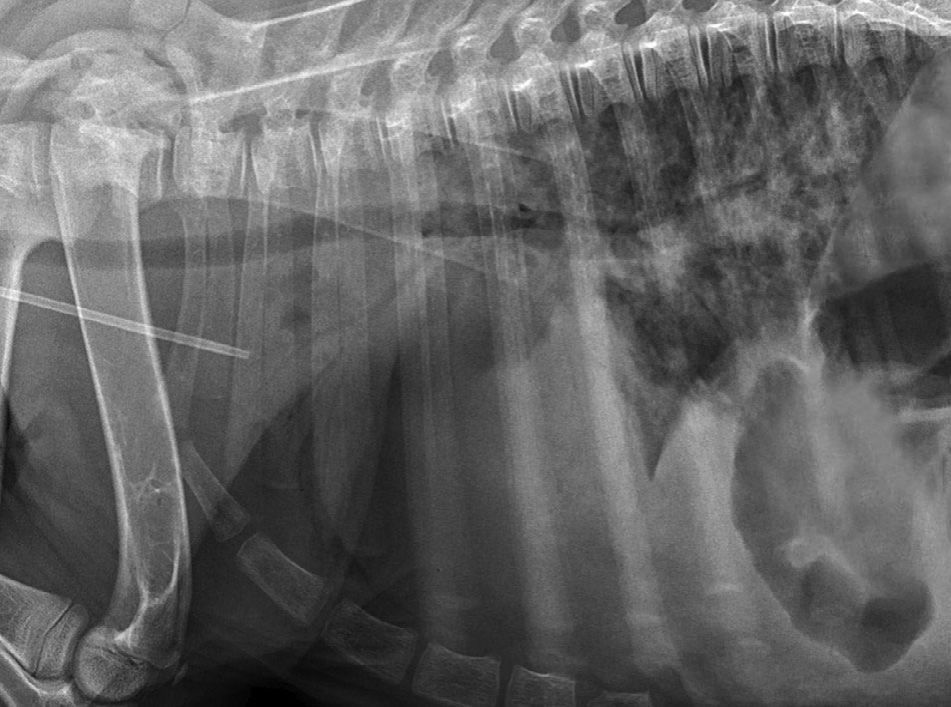
A repeat right lateral thoracic radiograph of an American Miniature Horse colt within 48 h of admission (4 days of age) for respiratory distress shows a progressive alveolar pattern in the ventral aspect of the caudal lung and a new alveolar pattern in the cranioventral lung, with evidence of air bronchograms. A marked mixed pulmonary pattern is noted in the caudodorsal lung fields. The cardiac silhouette appears normal, although evaluation is limited due to superimposition with the pulmonary changes.

Repeat thoracic radiographs at this time showed diffuse pulmonary infiltrates (Figure 5), consistent with significant disease progression. Thoracic POCUS revealed coalescing B‐lines, pleural irregularities, and areas of consolidation within the right mid‐hemithorax (Figure S1).

#### 3.2.2 Application of Domain 2

The patient now meets the five criteria outlined in the updated ARDSVet definition (Working Group Recommendations, Table 3):

1. **Risk Factor**: Aspiration injury, suspected sepsis
2. **Timing**: Respiratory distress within 1 week of perinatal illness
3. **Origin of Edema**: Left‐sided congestive heart failure and fluid overload were ruled out based on normal radiographic heart size, absence of cardiac history, heart murmur, or clinical signs, and conservative fluid therapy
4. **Thoracic Imaging**: Thoracic radiographs show diffuse pulmonary infiltrates, supporting ARDS due to increased permeability edema, and thoracic POCUS coalescing B‐lines, pleural irregularities and areas of consolidation within the right mid hemithorax.
5. **Oxygenation**: PaO_2_/FiO_2_ ratio of 120 (PaO_2_ 96 mm Hg, HFNO FiO_2_ 80%); mild/moderate hypoxemia ARDSVet category of non‐intubated ARDS

**TABLE 3:** The updated ARDSVet Definitions

Criteria that apply to all ARDSVet categories	
**Risk factors**	Precipitated by a known or suspected acute predisposing risk factor or clinical insult
**Origin of edema**	Left‐sided congestive heart failure (L‐CHF) and fluid overload as the cause of pulmonary infiltrates should be ruled out when diagnosing ARDS. Ultrasound (echocardiography, POCUS) is useful; however, thoracic radiography, history, and physical examination findings may be used to support ruling out L‐CHF and fluid overload
**Timing**	New or worsening respiratory distress within 1 week of known or suspected clinical insult
**Thoracic imaging**	Thoracic imaging demonstrating diffuse pulmonary infiltrates using CT, radiography, or thoracic ultrasound^a^ is required.
Airway fluid**	** ***Optional Supporting Criterion***: Neutrophilic inflammation and high protein levels in airway fluid collected through tracheal wash or bronchoalveolar lavage.

Criteria that apply to specific ARDSVet categories			
		IMV‐ARDS^d^	Non‐intubated ARDS
**Oxygenation** ^c^, ^e^	**Severity** **↓**	*Intubated, mechanically ventilated patients with PEEP of 5 cm H_2_O or higher* ***AND*** *must fulfill one of the criteria below*	*Equines >24h old: PaO_2_ ≤ 60 mm Hg*
			*Equines <24h old: PaO_2_ ≤ 45 mm Hg*
			***For all animals receiving supplemental oxygen***:
			**HFNO**: flow rate >1 L/kg/min or total >30 L/min OR supplemental oxygen with known FiO_2_ ***WITH***:
	***Mild/Moderate***	PaO_2_:FiO_2_ ratio > 100 and ≤ 300 ***OR***	
		SpO_2_/FiO_2_ ratio^b^ > 150 and ≤ 315 (with SpO_2_ ≤ 97%)	
	***Severe***	PaO_2_:FiO_2_ ratio ≤ 100 ***OR***	
		SpO_2_/FiO_2_ ratio^b^ ≤ 150 (with SpO_2_ ≤ 97%)	

Abbreviations: ARDS = acute respiratory distress syndrome; CT = computed tomography; HFNO = high‐flow nasal oxygen; IMV = invasive mechanical ventilation; L‐CHF = left‐sided congestive heart failure; PEEP = positive end‐expiratory pressure; POCUS = point of care ultrasound; SpO_2_ = oxygen saturation as measured by pulse oximetry

^a^ Thoracic radiographs or CT are preferred; however, ultrasound can be considered if radiographs or CT are not available. The ultrasound operator should be well trained in the use of ultrasound for identifying loss of lung aeration (e.g., multiple B lines and/or consolidations) and other ultrasound findings suggestive of noncardiogenic pulmonary edema (e.g., pleural line abnormalities).

^b^ Modified oxygenation criteria can be applied in settings in which arterial blood gas and/or HFNO, and mechanical ventilation are not routinely available.

^c^ For pulse oximetry, ensure an adequate waveform and oximeter placement. SpO_2_/FiO_2_ ratio is not valid above saturation values of 97%. Pulse oximetry is not recommended for diagnosis if a hemoglobin abnormality is suspected (e.g., methemoglobinemia or carboxyhemoglobinemia).

^d^ For all severity categories of IMV ARDS, a minimum PEEP of 5 cm H_2_O is required. Patients may move from one category to another throughout their disease course.

^e^ If altitude is >1,000m, apply the following correction factor: (PaO_2_ or SpO_2_)/FiO_2_ x (barometric pressure/760)

### 3.3 Domain 3: Advanced Respiratory Support Therapies in ARDS

#### 3.3.1 Application of Domain 3

At the time when HFNO was initiated, the colt met the oxygenation criterion for nonintubated ARDS based on a PaO_2_/FiO_2_ ratio of 120, consistent with mild/moderate hypoxemia (PaO_2_:FiO_2_ ratio >100 and ≤300, on HFNO with a flow rate of >1 L/kg/min; Working Group Recommendations, Table 3). The clinical, imaging, and oxygenation findings were thus sufficient to fulfill the criteria for nonintubated ARDS (Working Group Recommendations 2.3b‐d).

Supportive therapies, including intravenous antimicrobials, bronchodilators, hydrocortisone, and sildenafil, were initiated. The colt's respiratory rate and effort gradually improved over the course of hospitalization, though he still displayed mildly increased respiratory effort while recumbent. HFNO therapy was weaned over time through reduction in flow rate and FiO_2_. Thoracic radiographs and oxygenation normalized by day 14, after which the colt was discharged for further at‐home care.

#### 3.3.2 Summary

This case illustrates the importance of key updates in the new ARDSVet definitions, which allow classification of “at risk” patients in the early course of disease, to facilitate close monitoring and strategic intervention based on changes in clinical, imaging, and oxygenation characteristics. It demonstrates how patients may transition from “at risk” states to a diagnosis of ARDSVet and be characterized based on severity of hypoxemia, without requiring intubation.

ARDS severity categories now reflect the use of advanced yet noninvasive respiratory support, such as HFNO, without requiring access to these interventions for diagnosis, as oxygenation characteristics can still be met based on arterial blood gas analysis on room air. In newborn foals, hypoxemia may be stratified based on patient age (equids >24‐h old: PaO_2_ ≤60 mm Hg, equids <24‐h old: PaO_2_ ≤45 mm Hg; Working Group Recommendations, Table 3) and severity of hypoxemia into mild/moderate and severe ARDS without requiring intubation.

## 4 Conclusions

The ARDSVet definitions provide key updates to facilitate the recognition of ARDS in veterinary patients. These case vignettes illustrate the practical application of these definitions to aid in the early recognition and diagnosis of ARDS.

## Disclosure

The ARDSVet definitions referenced in this manuscript were presented at the Annual Scientific Symposium of the Veterinary Comparative Respiratory Society in Providence, RI in October 2023. Draft definitions were presented at the International Veterinary Emergency and Critical Care Symposium in St. Louis, MO in September 2024, and at the Dorothy Russell Havemeyer Consensus Statement Workshop on ARDS in Clearwater Beach, FL in October 2024.

## Ethics Statement

The authors confirm that the ethical policies of the journal, as noted on the journal's author guidelines page, have been adhered to. No ethical approval was required as this is a supplement to a review article with no original research data.

## Conflicts of Interest

The authors declare no conflicts of interest.

## Supporting information




**Supporting Figure 1**: Cine loop of a thoracic POCUS study of a colt with nonintubated ARDS demonstrates coalescing B‐lines, pleural irregularities, and areas of consolidation within the right mid‐hemithorax.
